# Exercise Lowers Plasma Angiopoietin-Like 2 in Men with Post-Acute Coronary Syndrome

**DOI:** 10.1371/journal.pone.0164598

**Published:** 2016-10-13

**Authors:** Nathalie Thorin-Trescases, Doug Hayami, Carol Yu, Xiaoyan Luo, Albert Nguyen, Jean-François Larouche, Julie Lalongé, Christine Henri, André Arsenault, Mathieu Gayda, Martin Juneau, Jean Lambert, Eric Thorin, Anil Nigam

**Affiliations:** 1 Montreal Heart Institute, Research Center, University of Montreal, Montreal, Quebec, Canada; 2 Cardiac Rehabilitation and Prevention Center (EPIC) of the Montreal Heart Institute, University of Montreal, Montreal, Quebec, Canada; 3 Departments of Pharmacology and Surgery, Faculty of Medicine, University of Montreal, Montreal, Quebec, Canada; 4 Montreal Behavioral Medicine Centre, Montreal, Quebec, Canada; 5 School of Public Health, University of Montreal, Montreal, Quebec, Canada; Department of Cardiology and Angiology, GERMANY

## Abstract

Pro-inflammatory angiopoietin-like 2 (angptl2) promotes endothelial dysfunction in mice and circulating angptl2 is higher in patients with cardiovascular diseases. We previously reported that a single bout of physical exercise was able to reduce angptl2 levels in coronary patients. We hypothesized that chronic exercise would reduce angptl2 in patients with post-acute coronary syndrome (ACS) and endothelial dysfunction. Post-ACS patients (n = 40, 10 women) were enrolled in a 3-month exercise-based prevention program. Plasma angptl2, hs-CRP, and endothelial function assessed by scintigraphic forearm blood flow, were measured before and at the end of the study. Exercise increased *V*O_2_peak by 10% (p<0.05), but did not significantly affect endothelial function, in both men and women. In contrast, exercise reduced angptl2 levels only in men (-26±7%, p<0.05), but unexpectedly not in women (+30±16%), despite similar initial levels in both groups. Exercise reduced hs-CRP levels in men but not in women. In men, levels of angptl2, but not of hs-CRP, reached at the end of the training program were negatively correlated with *V*O_2_peak (r = -0.462, p = 0.012) and with endothelial function (r = -0.419, p = 0.033) measured at baseline: better initial cardiopulmonary fitness and endothelial function correlated with lower angptl2 levels after exercise. Pre-exercise angptl2 levels were lower if left ventricular ejection time was long (p<0.05) and the drop in angptl2 induced by exercise was greater if the cardiac output was high (p<0.05). In conclusion, in post-ACS men, angptl2 levels are sensitive to chronic exercise training. Low circulating angptl2 reached after training may reflect good endothelial and cardiopulmonary functions.

## Introduction

Multiple evidence show that chronic physical exercise, as a primary or secondary prevention, is beneficial: this is illustrated by significant decrease in all-cause mortality [1–3] and in the risk of cardiovascular diseases (CVD) [2–5]. Chronic exercise slows the progression of coronary artery disease (CAD) as it preserves/improves both myocardial perfusion [6, 7] and vascular endothelial function [8, 9]. Chronic exercise also reduces oxidative stress [6, 9] and is anti-inflammatory by lowering the production of pro-inflammatory cytokines and proteins such as interleukins, adhesion molecules, fibrinogen and/or hs-CRP [1–4, 9–13]. Recently, we reported that in CAD patients, a single bout of exercise was able to lower circulating levels of angiopoietin-like 2 (angptl2) [14], a pro-inflammatory and pro-oxidative adipokine [15, 16].

Belonging to the angiopoietin-like family, angptl2 has been recently reported to contribute to chronic inflammation associated with atherosclerosis [17, 18], insulin resistance and obesity [19, 20] and multiple types of cancer (for review, [15, 16]). Accordingly, circulating levels of angptl2 are elevated in patients with CAD [14, 17, 18, 20, 21], diabetes, insulin resistance and obesity [19, 22] and cancer [23, 24]. Recently, elevated circulating levels of angptl2 were also reported in patients with post-acute coronary syndrome (ACS) [25] and it is now proposed that angptl2 is a risk factor for CVD [26]. Preclinical studies have indeed established that angptl2 induces vascular inflammation, including in endothelial cells and promote atherosclerosis [17, 18, 20]; however, its contribution to the development of chronic endothelial/vascular inflammation leading to endothelial dysfunction is less understood. We, and others, reported that angptl2 significantly reduced acetylcholine-induced dilation of isolated mouse arteries and, conversely, that angptl2 knockdown mice were protected against endothelial dysfunction induced by a high-fat diet [18, 27]. In human, information concerning the impact of angptl2 on endothelial function is scarce. A study performed in overweight but otherwise healthy Japanese men reported that a 3-month lifestyle intervention combining nutritional counseling and physical training reduced angptl2 plasma levels, in parallel with weight loss and improvement in lipid metabolism [28]. In contrast, we showed that although a single bout of exercise was able to reduce circulating levels of angptl2 in CAD patients, levels of angptl2 were low and unaffected by exercise in lean healthy physically active volunteers [14]. It remains to be determined whether chronic exercise, in patients with risk factors for CVD and endothelial dysfunction, would translate into a reduction in circulating levels of angptl2. Since angptl2 level is negatively correlated with *V*O_2_max [14] and since physical training improves cardiopulmonary fitness, we hypothesized that chronic aerobic exercise would lower angptl2 in post-ACS patients with endothelial dysfunction.

## Material and Methods

### Participants

In the context of a study aiming at evaluating the effects of 3 months of aerobic exercise training, 40 patients (30 men and 10 women) with post-ACS and optimally treated were recruited at the cardiovascular prevention center of the Montreal Heart Institute. Baseline characteristics and the medication of all subjects are presented in S1 Table. Post-ACS patients were hypertensive (25/40, 63%), diabetic (4/40, 10%), dyslipidemic (33/40, 83%), obese (25/40, 63%), smokers (5/40, 13%) or ex-smokers (23/40, 58%), and were new members of the cardiovascular prevention center. The mean duration after ACS was 65±7 days. The research protocol was approved by the Research Ethics and New Technology Development Committee of the Montreal Heart Institute. The study was registered on the site Clinical Trials (NCT02048696: Effect of exercise training on left ventricular function in patients post-myocardial infarction (EXIT-V)). Informed written consent was obtained from all individual participants (n = 40) included in the study. Selection of the patients, inclusion and exclusion criteria of the patients have been previously reported [29]. Blood, anthropometric, fitness and hemodynamic parameters were measured before and after the training program in n = 40 post-ACS patients. Among them, endothelial function was measured in 31 patients.

### Experimental design

Before and at the end of the 3-month aerobic exercise training program, all patients underwent a complete medical evaluation and a maximal cardiopulmonary exercise test on an electromechanically braked bicycle ergometer, according to previous methodology [30], which allowed the measurement of maximal oxygen uptake (*V*O_2_peak, ml.min^-1^.kg^-1^ of lean body mass), blood pressure and heart rate. During the 3 months (36 sessions of training), all subjects performed aerobic exercise under the supervision of an exercise physiologist, a nurse, and a cardiologist. The aerobic exercise training program was realized on a bike (Precor, model 846i, USA). A 5 min warm-up at 30% of *V*O_2_peak was perfomed followed by a 40-min of cycling between 60 to 80% of *V*O_2_peak and finally, a 5-minute recovery period at 30% of *V*O_2_peak was realized. The exercise intensity was gradually increased during the program according to patient’s tolerance and rate of perceived exertion (Borg scale level between 11 to 15). Total time duration of each session was around 50 minutes.

### Measurement of hemodynamic parameters

Cardiac bioimpedance (PhysioFlow, Enduro model, Manatec, France) was used to measure central hemodynamic modifications during the maximal cardiopulmonary exercise test before and after the aerobic exercise training program [31]. This non-invasive technique was found to be valid, accurate, and reproducible at rest and during exercise in healthy subjects and cardiac patients [32, 33]. Among other parameters, cardiac output (CO, L/min) and left ventricular ejection time (LVET, ms) were measured with this device on a beat-to-beat basis and were then averaged every 15 sec for data analysis.

### Measurement of endothelial function

Endothelial function was measured non-invasively by quantitative scintigraphic imaging of hyperemic reactivity in the right arm, as previously described [34]. Briefly, after 5 min of right arm ischemia, the nuclear tracer technetium-99m-tetrofosmin (Myoview) was injected in the antebrachial vein of the left arm through an indwelling catheter after one-minute delay to allow the capture of sustained nitric oxide-dependent hyperemic response. Upon scanning, the activity-time curves of the hyperemic right forearm and that of the contra-lateral left forearm were analyzed. The peak slopes of the initial activity-time curves were calculated in the right and left arm. The ratio of the slopes in the right-to-left arm was used as an index of endothelial function: the higher the ratio, the better endothelial function. A right-to-left slope ratio lower than 3.55 has been reported to be a cutoff value indicative of endothelial dysfunction [34].

### Laboratory analyses

Venous blood samples were collected before and at the end of the 3-month training, centrifuged and plasma was stored at -80°C until further analysis. Fasting circulating levels of angptl2 were measured using a commercial ELISA kit (#ABIN 415096; antibodies-online.com), as previously described [14, 17, 29]. Fasting levels of glucose, insulin, triglycerides, total cholesterol, HDL- and LDL-cholesterol and hs-CRP were assessed by the clinical biochemical laboratory of the Montreal Heart Institute.

### Statistical analysis

Continuous normally distributed variables are presented as means and standard errors. Continuous non-normally distributed variables are presented as median and [25^th^ - 75^th^ percentiles]. Categorical variables are presented as frequencies and percentages. Normal Gaussian distribution of the data was verified by the d'Agostino and Pearson omnibus normality test. If the data were not normally distributed, data were transformed in a first step to keep the maximum of information and to maximize the power of testing, and if still not normally distributed, non-parametric tests were used. The changes in angptl2 levels between groups (Men *versus* Women) were compared using a one-way analysis of covariance, controlling for baseline values. To determine the impact of individual cardiovascular risk factors on the continuous parameters measured (angptl2 and hs-CRP levels) multiple linear regressions were used. A stepwise approach was used to select risk factors. Goodness of fit was checked with usual diagnosis statistics such as studentized deleted residuals, leverage values and Cook’s distances. The significance level was set at 0.05. Statistical analyses were performed with SPSS, V22, SPSS Inc., Chicago, USA.

## Results

### Impact of exercise on anthropometric, hemodynamic, and fitness parameters in post-ACS patients

As expected, women had a lower body mass and a higher fat mass % when compared to men before and after exercise (Table 1). Overall, body mass, lean and fat mass, waist circumference and BMI, resting heart rate, resting systolic and diastolic pressures were not affected by the 3-month exercise training in post-ACS patients (Table 1), regardless of the sex of the patient (Table 1). Nevertheless, compliance to the training program was demonstrated by a significant increase in *V*O_2_peak in both men (from 28.4 [25.1–33.6]) to 33.8 [26.7–38.0] ml/min/kg of lean body mass, n = 29, P = 0.0010) and women (from 25.8±1.0 to 28.6±1.5 ml/min/kg of lean body mass, n = 10, P = 0.0033) (Table 1). Compliance to the training program was similar between men (98.9±3.0%) and women (97.0±3.4%).

**Table 1:** Impact of 3-month aerobic exercise training on anthropometric and hemodynamic parameters in post-acute coronary syndrome patients (data are mean±SEM or median [25^th^-75^th^] of (n) patients).

	Post-ACS patients (n = 40)	Post-ACS Men (n = 30)	Post-ACS Women (n = 10)	p-value Men *versus* Women
**Body mass (kgs)**				
Baseline	81.6±2.4 (40)	85.2±2.6 (30)	70.8±3.4 (10)	**0.0066**
After exercise	81.6±2.4 (40)	85.2±2.8 (30)	70.8±3.7 (10)	**0.0089**
**Waist circumference (cm)**				
Baseline	99.2±1.8 (36)	97.5 [94–108] (28)	93.1±4.2 (8)	**0.0360**
After exercise	99.8±2.1 (32)	99.0 [94–105] (25)	96.8±6.8 (7)	0.2091
**Fat mass (%)**				
Baseline	28.5±1.2 (39)	25.2±1.1 (29)	37.8±1.3 (10)	**<0.0001**
After exercise	28.1±1.3 (40)	24.7±1.1 (30)	37.6±1.5 (10)	**<0.0001**
**Trunk fat mass (%)**				
Baseline	29.0±1.0 (39)	27.3±1.2 (29)	34.0±1.1 (10)	**0.0036**
After exercise	28.5±1.2 (40)	26.5±1.3 (30)	34.9±1.8 (10)	**0.0018**
**BMI (kg/m**^**2**^**)**				
Baseline	26.9 [25–31] (40)	27.4 [25–30] (30)	28.6±1.4 (10)	0.8759
After exercise	26.9 [25–30] (40)	26.7 [25–30] (30)	28.6±1.5 (10)	0.7906
**Resting heart rate (bpm)**				
Baseline	64±1 (40)	65±2 (30)	63±2 (10)	0.6344
After exercise	64±1 (40)	64±1 (30)	64±2 (10)	0.9056
**Resting SBP (mm Hg)**				
Baseline	120 [110–130] (40)	118 [110–129] (30)	129±4 (10)	0.0537
After exercise	118 [108–130] (40)	117 [108–124] (30)	132±7 (10)	0.0877
**Resting DBP (mm Hg)**				
Baseline	69±1 (40)	69±1 (30)	70±3 (10)	0.7941
After exercise	68±1 (40)	68±1 (30)	72±2 (10)	0.0898
***V*O**_**2**_**max/LBM (ml/min/kg)**				
Baseline	28.3 [25–32] (39)	28.4 [25–34] (29)	25.8±1.0 (10)	0.0644
After exercise	32.2 [26–137] * (40)	33.8 [27–38] * (30)	28.6±1.5 * (10)	**0.0456**

*: p<0.05 versus baseline

BMI: body mass index; SBP: systolic blood pressure; DBP: diastolic blood pressure; *V*O_2_max/LBM: maximal cardiorespiratory capacity corrected by the lean body mass.

### Impact of sex on circulating angptl2 levels in post-ACS patients

**Fig 1:**
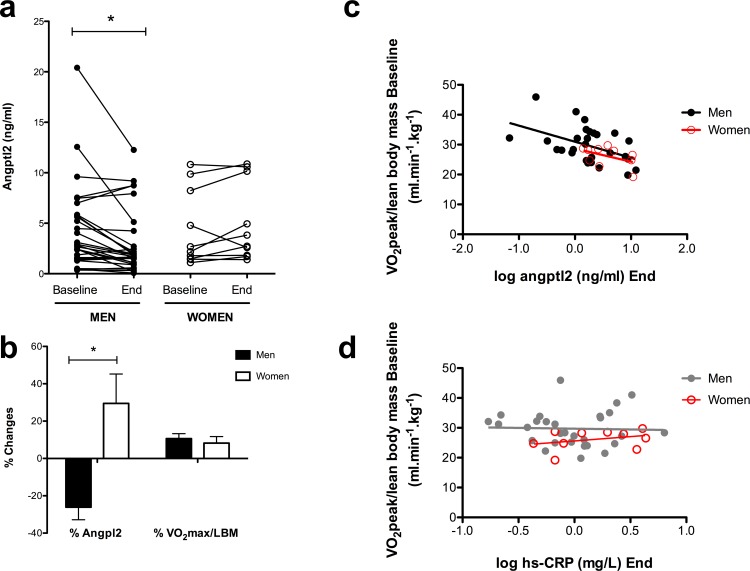
(**a**) Three-month aerobic exercise training lowered circulating angptl2 levels in post-acute coronary syndrome men (n = 30), but not in women (n = 10). Angptl2 levels were measured at baseline and at the end of the 3-month prevention program. Data are presented as paired individual values; *: P<0.05 *versus* baseline (Wicolxon signed rank test). (**b**) Percentage changes in angptl2 levels and in cardiopulmonary fitness (*V*O_2_peak/lean body mass, ml/min/kg) between baseline and the end of the training program. Negative values correspond to a reduction in angptl2 levels; positive values correspond to an increase in angptl2 levels or in *V*O_2_peak. Data are presented as mean±SEM of n values; *: P<0.05 *versus* values observed in men (Mann-Whitney test). (**c**) Negative correlation between angptl2 levels (log transformed) measured at the end of the 3-month training program and *V*O_2_peak measured at baseline (in men: r = -0.462, P = 0.0116, n = 29; in women: r = -0.461, P = 0.1797, n = 10). (**d**) No correlation between hs-CRP levels (log transformed) measured at the end of the 3-month training program and *V*O_2_peak measured at baseline (in men: r = -0.033, P = 0.865, n = 29; in women: r = 0.330, P = 0.352, n = 10).

There was no difference in baseline angptl2 levels between men and women (Table 2), as previously reported by us [14] and others [20, 35]. Unexpectedly, at the end of the 3-month program, final angptl2 levels were different between men and women: in male post-ACS patients, exercise led to lower angptl2 levels than in female patients (Table 2). Accordingly, delta angptl2 levels (angptl2 levels at baseline—angptl2 at the end of the program) were significantly different between men and women (Table 2). Altogether, these data show that 3 months of physical aerobic training significantly decreased circulating angptl2 levels in male, but not in female post-ACS patients (Fig 1A). Despite a similar increase in *V*O_2_peak in both groups, the effect of chronic aerobic exercise on circulating angptl2 levels was therefore opposite in men (reduction of angptl2 by -26±7%, p<0.05) and women (non significant change of angptl2 by +30±16%) (Fig 1B).

**Table 2:** Effect of sex on initial, final and initial-final (Delta) circulating levels of angptl2 (ng/ml) (mean±SEM or median [25^th^-75^th^] of (n) patients). P values for the effect of sex on angptl2 values are indicated.

	Angptl2_initial_	Angptl2_final_	Delta Angptl2_initial_—Angptl2_final_
Men	2.75 [1.51–5.77] (30)	1.68 [1.02–3.09] (30)	0.41 [-0.08–2.31] (30)
Women	4.44±1.20 (10)	5.06±1.24 (10)	-0.63±0.43 (10)
P value Sex	0.864	0.030	0.006

### Relationship between angptl2 levels and VO_2_peak

Levels of angptl2 measured at the end of the program were negatively correlated with baseline *V*O_2_peak in men (r = -0.462, p = 0.0116, n = 29) and to a lesser extent in women (r = -0.461, p = 0.1797, n = 10): the fitter the patient, the lower angptl2 levels after 3 months of exercise (Fig 1C), suggesting that a low angptl2 level promoted by exercise training is associated with a better cardiopulmonary fitness. In contrast, there was no significant correlation between *V*O_2_peak and hs-CRP levels, neither in men (r = -0.033, P = 0.865, n = 29) nor in women (r = 0.330, p = 0.3522, n = 10) (Fig 1D).

### Impact of exercise on hs-CRP levels and other blood markers

Globally, chronic exercise training decreased hs-CRP levels by 30% (S2 Table). Exercise lowered hs-CRP in men (P = 0.0221), but not in women (P = 0.3165, S2 Table). Glucose, insulin, total cholesterol, HDL-cholesterol, LDL-cholesterol and triglycerides levels were not affected by the training program, regardless of the sex (S2 Table).

### Impact of aerobic exercise training on endothelial function

**Fig 2:**
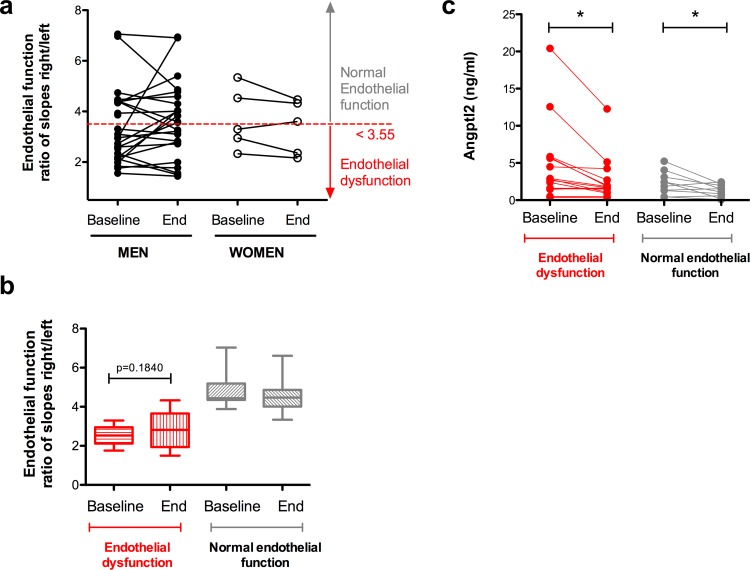
(**a**) Three-month aerobic exercise training affected endothelial function neither in men (n = 26) nor in women (n = 5) with post-acute coronary syndrome. Endothelial function was measured at baseline and at the end of the program. (**b**) Endothelial function before and after exercise in patients (men and women combined) categorized according to their initial value of endothelial function: endothelial dysfunction is defined by baseline values lower than 3.55. Data are presented as paired individual values in (**a**) and as median [10^th^-90^th^] in (**b**). (**c**) Aerobic exercise training reduces angptl2 levels in post-acute coronary syndrome men patients displaying baseline endothelial dysfunction (defined with values of endothelial function lower than 3.55) (n = 15), or normal endothelial function (n = 9). Angptl2 levels were measured at baseline and at the end of the program. Data are presented as paired individual values. *: P<0.05 *versus* baseline (Wicolxon signed rank test).

Endothelial function was assessed before and at the end of the training program: 3 months of physical aerobic training did not improve endothelial function in either male or female post-ACS patients (Fig 2A). Of note, all patients at baseline did not exhibit endothelial dysfunction (defined by values of the ratio of the slopes right-to-left arm lower than 3.55), but even in patients with initial endothelial dysfunction (19/33 patients), 3 months of exercise had no statistically significant (P = 0.1840) impact on endothelial function (Fig 2B), either in men or in women (data not shown).

### Relationship between angptl2 levels and endothelial function

**Fig 3:**
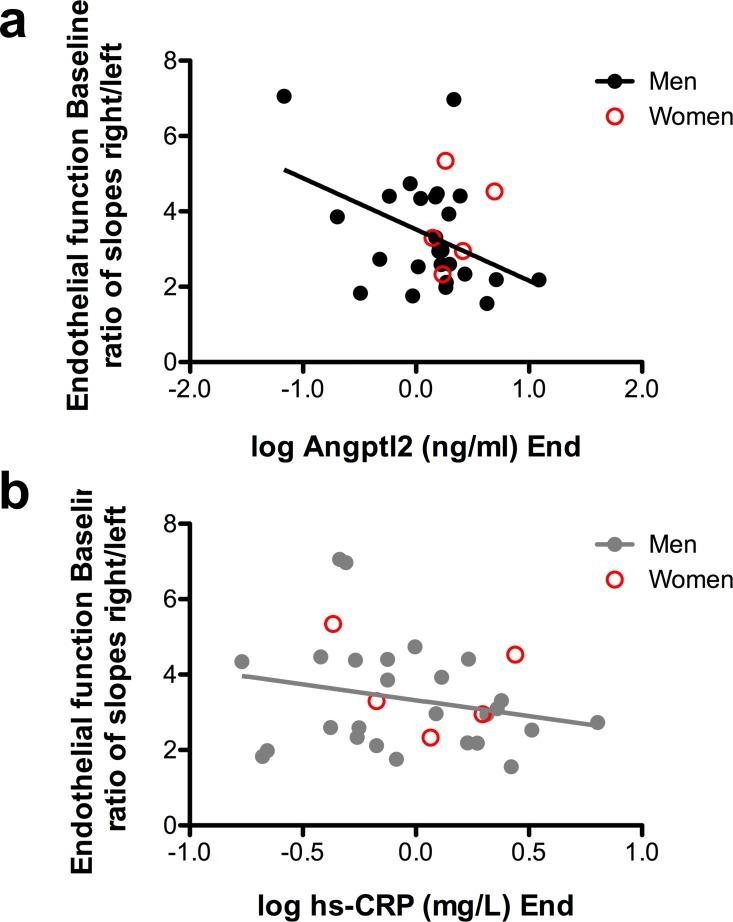
(**a**) Negative correlation between angptl2 levels (log transformed) measured at the end of the training program and endothelial function measured in baseline (in men: r = -0.419, P = 0.0031, n = 26). (**b**) No correlation between hs-CRP levels (log transformed) measured at the end of the training program and baseline endothelial function (in men: r = -0.228, P = 0.2623, n = 26).

Before the training program, in patients (men and women) with initial endothelial dysfunction, basal plasma angptl2 levels were not significantly higher than in patients with normal endothelial function (median 2.67 [1.46–5.64], n = 19 *versus* 2.02 [1.30–2.94], n = 12, P = 0.3011). If only men were considered, exercise reduced angptl2 levels in patients with or without endothelial dysfunction (Fig 2C). Interestingly, a negative correlation was found in men between endothelial function measured at baseline and the angptl2 levels reached after 3 months of training (r = -0.419, p = 0.0331, n = 26): the better the endothelial function at baseline, the lower the final angptl2 levels (Fig 3A and Table 3), suggesting that a low angptl2 level promoted by exercise training is associated with a better endothelial function. In contrast, there was no significant correlation between endothelial function and hs-CRP levels, neither in men (r = -0.228, P = 0.2623, n = 26) nor in women (Fig 3B).

**Table 3:** Regression coefficient (B), standard error and P values for the association between different independent variables on initial, final and initial-final circulating levels of angptl2 (Ln transformed).

Dependent variable	Independent variable	B	Standard error	P value
Ln Angptl2_initial_	LVET_initial_	-0.003	0.001	0.007
Ln Angptl2_final_	Endothelial function_initial_	-0.146	0.069	0.048
LnAngptl2initial−LnAngptl2_final_	Cardiac output_initial_	0.031	0.014	0.032

### Impact of cardiovascular risk factors on angptl2 levels

Risk factors such as hypertension, dyslipidemia, diabetes, smoking and obesity had no significant impact on angptl2 levels, measured either at baseline or at the end of the training program (data not shown).

### Impact of cardiac function indexes on angptl2 levels

A negative association was found between the angptl2 levels measured at baseline and the left ventricular ejection time (LVET): a longer LVET was slightly, but significantly, associated with lower basal circulating angptl2 (Table 3). In addition, a positive association was found between the delta angptl2 levels (Ln angpl2 levels at baseline—Ln angptl2 at the end of the program) and the cardiac output measured at baseline: a strong initial cardiac output increased slightly the reduction in angptl2 levels observed after 3 months of training (Table 3). Altogether, these data suggest, for the first time, a potential link between cardiac function and circulating angptl2 levels. However, the other cardiac parameters calculated in patients, such as the stroke volume, the ejection fraction, the contractility index, the end diastolic volume, and the systemic vascular resistance had no significant impact on angptl2 levels, measured either at baseline or at the end of the training program (data not shown).

## Discussion

The major findings of this study are that 3 months of aerobic exercise training reduce elevated angptl2 circulating levels in post-ACS patients. We found, however, that while exercise significantly lowered angptl2 levels in post-ACS men, the same training seemed to have no effect in post-ACS women. A long LVET and a high CO were associated with beneficial effects of exercise on angptl2. Finally, and most interestingly, basal endothelial function and basal *V*O_2_max measured in men were negatively correlated with angptl2 levels reached after 3 months of exercise: the better the endothelial function and the fitter the patient, the lower angptl2 levels. Altogether, these data suggest for the first time that circulating angptl2 levels are sensitive to chronic physical training in post-ACS men and that angptl2 could be a marker of endothelial function and cardiopulmonary fitness.

Because angptl2 is pro-inflammatory and accelerates the atherosclerotic process [16–18, 20], any intervention capable of reducing abnormally high levels of angptl2 should be beneficial. A study performed in overweight but otherwise healthy Japanese men reported that a 3-month lifestyle intervention combining nutritional counseling and physical training reduced angptl2 plasma levels, in parallel with weight loss and improvement in lipid metabolism [28]. In this latter study, the modalities of exercise were not mentioned and the effect of exercise on angptl2 levels could not be deciphered from the effect of the diet. Nevertheless, the data suggested that chronic exercise was effective at lowering levels of circulating pro-inflammatory angptl2 in overweight men [28]; data in overweight women were not available. We recently reported that one single bout of exercise was able to lower angptl2 levels in CAD (13 men and 1 woman) patients, but not in healthy controls (20 men and 20 women) in whom angptl2 levels were low and unaffected by exercise [14]. Unexpectedly, we observed in the present study that chronic physical training significantly reduced plasma angptl2 in post-ACS men, but not in women. In fact, angptl2 levels tended to increase in post-ACS women after 3 months of exercise (Fig 1). Despite the low number of women included in this study (n = 10), statistical analysis showed a significant sexual dimorphism in the impact of chronic exercise on angptl2 levels in post-ACS patients. While it is clear that there are inherent anthropometric and physiological differences between men and women in responses to exercise [36], our data show for the first time that exercise may have an opposite effect on angptl2 according to sex. There is a real paucity of data concerning the effects of exercise in women and little is known about plasma angptl2 regulation. It is known that exercise reduces angptl2 levels [28], that epinephrine–which is released during exercise–inhibits angptl2 expression [22], but the link between exercise-induced increased catecholamines and exercise-induced decreased angptl2 has not been established. Interestingly, it has been reported by some that catecholamines concentrations are higher in men than in women during exercise [37–39], while others observed no sexual difference in the sympathetic drive induced by exercise [40]. Thus, a higher concentration of exercise-induced epinephrine in men could, in theory, promote a stronger reduction in angptl2 levels, as observed in our study. This is only hypothetical and remains to be demonstrated. While it must be confirmed that exercise does not lower angptl2 in post-ACS women, our study clearly shows that chronic exercise reduces angptl2 in post-ACS men, extending the work of Muramoto et al. performed in healthy overweight men [28] and our previous study assessing the effect of acute exercise on angptl2 in CAD patients [14].

We observed that both a long LVET and a high CO were associated with beneficial effects of exercise on angptl2: longer LVET was associated with lower pre-exercise angptl2 levels and higher CO was associated with a larger reduction in angptl2 induced by chronic training (Table 3). To the best of our knowledge, this is the first report of an association between angptl2 and cardiac function: while angptl2 contributes to various vascular chronic inflammatory diseases (for review, [15, 16]), its role in cardiac physiology or pathology is unknown. A long LVET and a high CO could reflect a "healthy" cardiac function, and thus explain the beneficial effects of exercise on angptl2 in these post-ACS patients. However, although statistically significant, the impact of LVET or CO on angptl2 was minimal (Table 3); the physiological significance of these cardiac parameters on angptl2 levels remains to be further explored. We also did not find an impact of risk factors for CVD on angptl2 levels, although in humans, circulating angptl2 levels have been shown to be elevated in overweight or obese subjects and to correlate with inflammation and degree of insulin resistance [20, 22]. In addition, in a large study performed in the general Japanese population, it was reported that the risk of developing diabetes was higher in the subjects in the highest serum angptl2 quartile, demonstrating that serum angptl2 level is an independent risk factor for the development of type 2 diabetes [19]. In the present study, only 10% of the patients were diabetic, likely explaining the lack of association between diabetes and angptl2 levels.

We did not observe an improvement in endothelial function after 3 months of aerobic exercise, even in the patients (men or women) displaying an endothelial dysfunction at baseline (Fig 2). Baseline angptl2 levels were similar in patients with or without endothelial dysfunction, and exercise reduced angptl2 levels regardless of the endothelial function (Fig 2). Nevertheless, we report a negative relationship between circulating levels of angptl2 reached after 3 months of exercise and initial endothelial function, in men (Fig 3), suggesting that a low angptl2 level promoted by exercise training is associated with a better initial endothelial function. Similarly, we also report a negative relationship between circulating levels of angptl2 reached after 3 months of exercise and initial *V*O_2_max, in men (Fig 1), suggesting that a low angptl2 level promoted by exercise training is associated with a better initial cardiopulmonary function. Little is known about the impact of angptl2 on endothelial function and *vice versa*. We, and others, reported that recombinant angptl2 significantly reduced acetylcholine-induced dilation of isolated mouse arteries and, conversely, that angptl2 knockdown or knockout mice were protected against endothelial dysfunction induced by a high-fat diet [18, 27] and chronic infusion of low dose angiotensin II [41]. Altogether, these data suggest that angptl2 could be a marker of endothelial function. Remarkably, hs-CRP, a very commonly used inflammatory marker, was not correlated with either endothelial function or *V*O_2_max, making angptl2 a sensitive and interesting target for further investigation.

### Limitations of the study

The main limitation of the present study is the low number of women (n = 10, 25%) included in the training program. Three reasons may explain this low *n*: first, it is well known that myocardial infarction is more highly prevalent in men than in woman [42]. Second, this men (75%)/women (25%) ratio is similar to what we have generally observed in our laboratory, in previous studies on exercise regarding participation of women with heart diseases [30, 31]. Third, it is well documentated that women with coronary heart disease are generally less referred and/or participate less to cardiac rehabilitation program than men with coronary heart disease [43]. Another potential limitation of the study is the measurement of brachial artery endothelial function by an unconventional method (radionucleotide method instead of ultrasound method) and the fact that endothelial function was not measured in all patients (31/40). However, this method permits to assess endothelial dysfunction when the right-to-left slope ratio is lower than 3.55, a cutoff value indicative of endothelial dysfunction [34]. The strength of the study is that despite globally similar baseline characteristics between men and women, physical training clearly lowered Angptl2 levels in men, but not in women.

In conclusion, 3 months of aerobic exercise training reduced high pro-inflammatory angptl2 circulating levels in post-ACS patients, at least in men. Cardiopulmonary fitness and endothelial function were negatively correlated with angptl2 levels: the fitter the patient and the better the endothelial function, the lower angptl2. Altogether, these data suggest for the first time that circulating angptl2 levels are sensitive to chronic physical training in post-ACS men. More importantly, we propose that angptl2 could be a marker of endothelial function and more globally, a marker of cardiopulmonary fitness in patients with coronary artery diseases.

## Supporting Information

S1 Table Baseline parameters of the post-acute coronary syndrome patients. (DOCX)

S2 Table Impact of 3-month aerobic exercise training on blood parameters. (DOCX)
