# Supplementary material for: Exercise Lowers Plasma Angiopoietin-Like 2 in Men with Post-Acute Coronary Syndrome
Source: PLoS One. 2016 Oct 13;11(10):e0164598. doi: 10.1371/journal.pone.0164598 (PMC5063321; doi:10.1371/journal.pone.0164598)
Supplement: S1 Table — (DOCX) [file pone.0164598.s001.docx]

**Exercise lowers plasma angiopoietin-like 2 in men with post-acute coronary syndrome**

Nathalie Thorin-Trescases^1^, Doug Hayami^1,2^, Carol Yu^1,3^, Xiaoyan Luo^1^, Albert Nguyen^1,3^, Jean-François Larouche^1,2^, Julie Lalongé^2^, Christine Henri^1^, André Arsenault^1,4^, Mathieu Gayda^1,2^, Martin Juneau^1,2^, Jean Lambert^1,5^, Eric Thorin^1,3,6¶^*, Anil Nigam ^1,2,6¶^

**S1 Table:** Baseline parameters of the post-acute coronary syndrome patients (data are mean±SEM of n=40 patients, n=30 men and n=10 women)

|  | **Post-ACS patients (n=40)** | **Post-ACS Men (n=30)** | **Post-ACS Women (n=10)** | **p-value Men *versus* Women** |
| --- | --- | --- | --- | --- |
| **Age (years)** | 62±2 [35-74] | 60 ± 2 [35-74] | 68 ± 1 [63-74] | **0.0180** |
| **Actual PTCI** | 40/40 (100%) | 30/30 (100%) | 10/10 (100%) | 1.0000 |
| **Actual MI**  **LVEF (%)** | 36/40 (90%)  56.5±1.2 | 27/30 (90%)  57.3±1.4 | 9/10 (90%)  58.3±3.7 | 1.0000  0.7562 |
| **Family history** | 20/40 (50%) | 16/30 (53%) | 4/10 (40%) | 0.4652 |
| **Hypertension** | 25/40 (63%) | 17/30 (57%) | 8/10 (80%) | 0.1869 |
| **Type II diabetes** | 4/40 (10%) | 3/30 (10%) | 1/10 (10%) | 1.0000 |
| **Dyslipidemia** | 33/40 (83%) | 25/30 (83%) | 8/10 (80%) | 0.8101 |
| **Obesity** | 25/40 (63%) | 17/30 (57%) | 8/10 (80%) | 0.1869 |
| **Smoking** | 5/40 (13%) | 5/30 (17%) | 0/10 (0%) | 0.1675 |
| **Ex smoking** | 23/40 (58%) | 17/30 (57%) | 6/10 (60%) | 0.8535 |
| **Medication** |  |  |  |  |
| Statins | 39/40 (98%) | 29/30 (97%) | 10/10 (100%) | 0.5587 |
| Aspirin | 39/40 (98%) | 29/30 (97%) | 10/10 (100%) | 0.5587 |
| β-blockers | 34/40 (85%) | 25/30 (83%) | 9/10 (90%) | 0.6091 |
| Angiotensin Converting Enzyme inhibitors | 26/40 (65%) | 21/30 (70%) | 5/10 (50%) | 0.2508 |
| Clopidogrel/Pasugrel | 25/40 (63%) | 17/30 (57%) | 8/10 (80%) | 0.1869 |
| Nitrates | 19/40 (48%) | 14/30 (47%) | 5/10 (50%) | 0.8550 |
| Calcium channel blockers | 3/40 (8%) | 2/30 (7%) | 1/10 (10%) | 0.7289 |
| Angiotensin II receptor antagonists | 3/40 (8%) | 2/30 (7%) | 1/10 (10%) | 0.7289 |
| Diuretics | 1/40 (3%) | 0/30 (0%) | 1/10 (10%) | 0.0845 |

LVEF: left ventricular ejection fraction (measured by cardiac echography); MI: myocardial infarction; PTCI: percutaneous transluminal coronary angioplasty.

P-values for categories were assessed with Chi-square test with one degree of freedom.

P-value for LVEF was assessed with an unpaired t-test.
