# Supplementary material for: Exercise Lowers Plasma Angiopoietin-Like 2 in Men with Post-Acute Coronary Syndrome
Source: PLoS One. 2016 Oct 13;11(10):e0164598. doi: 10.1371/journal.pone.0164598 (PMC5063321; doi:10.1371/journal.pone.0164598)
Supplement: S2 Table — (DOCX) [file pone.0164598.s002.docx]

**Exercise lowers plasma angiopoietin-like 2 in men with post-acute coronary syndrome**

Nathalie Thorin-Trescases^1^, Doug Hayami^1,2^, Carol Yu^1,3^, Xiaoyan Luo^1^, Albert Nguyen^1,3^, Jean-François Larouche^1,2^, Julie Lalongé^2^, Christine Henri^1^, André Arsenault^1,4^, Mathieu Gayda^1,2^, Martin Juneau^1,2^, Jean Lambert^1,5^, Eric Thorin^1,3,6¶^*, Anil Nigam ^1,2,6¶^

**S2 Table:** Impact of 3-month aerobic exercise training on blood parameters in post-acute coronary syndrome men and women. Data are mean±SEM or median [25^th^-75^th^ percentiles] of (n) patients.

|  | **Post-ACS patients (n=40)** | **Post-ACS**  **Men (n=30)** | **Post-ACS Women (n=10)** | **p-value Men *versus* Women** |
| --- | --- | --- | --- | --- |
| **hs-CRP (mg/L)**  Baseline  After exercise | 1.0 [0.7-3.3] (38)  1.0 [0.6-2.0] * (40) | 1.0 [0.6-2.7] (29)  0.9 [0.5-1.8] * (30) | 3.0±1.0 (9)  2.0±0.5 (10) | 0.2717  0.1377 |
| **Glucose (mmol/L)**  Baseline  After exercise | 5.4 [5.0-5.8] (40)  5.2 [5.0-6.0] (39) | 5.5 [5.0-6.0] (30)  5.4 [5.1-6.0] (30) | 5.4±0.2 (10)  5.2±0.2 (9) | 0.4427  0.1807 |
| **Insulin (pmol/L)**  Baseline  After exercise | 56.0 [43-100] (39)  70.5 [49-111] (40) | 56.0 [41-101] (29)  71.0 [50-115] (30) | 75.6±10.6 (10)  73.2±14.5 (10) | 0.6180  0.4821 |
| **Total Cholesterol (mmol/L)**  Baseline  After exercise | 2.9±0.1 (40)  3.0±0.1 (40) | 2.9±0.1 (30)  3.0±0.1 (30) | 3.1 [2.7-3.2] (10)  3.2 [3.0-3.3] (10) | 0.5739  0.4534 |
| **Cholesterol-HDL (mmol/L)**  Baseline  After exercise | 1.0 [0.9-1.1] (40)  1.0 [0.9-1.2] (40) | 1.0 [0.9-1.1] (30)  1.0 [0.9-1.1] (30) | 1.1±0.1 (10)  1.2±0.1 (10) | 0.6282  0.1487 |
| **Cholesterol-LDL (mmol/L)**  Baseline  After exercise | 1.4±0.1 (40)  1.5±0.1 (40) | 1.4±0.1 (30)  1.5±0.1 (30) | 1.4±0.1 (10)  1.4±0.1 (30) | 0.8330  0.6352 |
| **Ratio total cholesterol/HDL**  Baseline  After exercise | 2.8 [2.5-3.3] (40)  2.8 [2.5-3.1] (40) | 2.9 [2.5-3.4] (30)  2.9 [2.4-3.2] (30) | 2.7±0.1 (10)  2.7±0.1 (10) | 0.3174  0.3733 |
| **Triglyceride (mmol/L)**  Baseline  After exercise | 1.0 [0.8-1.4] (40)  0.9 [0.8-1.3] (40) | 0.9 [0.8-1.3] (30)  0.9 [0.8-1.3] (30) | 1.1±0.1 (10)  1.0±0.1 (10) | 0.5846  0.1894 |
| **Ratio triglyceride/HDL**  Baseline  After exercise | 1.0 [0.8-1.4] (40)  0.9 [0.7-1.3] (40) | 1.0 [0.8-1.4] (30)  0.9 [0.7-1.3] (30) | 1.0±0.1 (10)  1.0±0.1 (10) | 0.9129  1.0000 |

*: p<0.05 *versus* baseline
